# Oropharynx HPV status and its relation to HIV infection

**DOI:** 10.7717/peerj.4407

**Published:** 2018-03-22

**Authors:** Leonora Maciel de Souza Vianna, Fabiana Pirani Carneiro, Rivadavio Amorim, Eliete Neves da Silva Guerra, Florêncio Figueiredo Cavalcanti Neto, Valdenize Tiziani, Andrea Barretto Motoyama, Anamélia Lorenzetti Bocca

**Affiliations:** 1 Department of Pathology, School of Medicine, University of Brasilia (UnB), Brasíla, DF, Brazil; 2 Department of Oral Pathology, School of Dentistry, University of Brasilia (UnB), Brasilia, DF, Brazil; 3 Center for Learning and Research, Brasilia Children Hospital, Brasilia-DF, DF, Brasil

**Keywords:** Acquired Immunodeficiency Syndrome, Oropharynx, Human Papillomavirus Test, Human Papillomavirus, HIV

## Abstract

**Background:**

The number of oropharyngeal lesions caused by HPV (Human papillomavirus) has been increasing worldwide in the past years. In spite of the clinical relevance of HPV infection in the anogenital tract of HIV-positive patients, the relevance of oropharynx HPV infection in these patients is not clear. The aim of the present study was to detect HPV infection, and clinical and cytological changes in the oropharynx of HIV-positive patients.

**Methods:**

Samples collected from the oropharynx of 100 HIV-positive patients were subjected to hybrid capture (HC), conventional and liquid-based cytology. Clinical data were also collected to investigate the relation with HPV status.

**Results:**

High and low-risk types of HPV were present in 8% and 16.7% of the total sample. The mean ± sd (maximum-minimum) of the relative ratio light unit (RLU)/cutoff (CO) was 2.94 ± 2.58 (1.09–7.87) and 1.61 ± 0.65 (1.07–2.8) for high- and low-risk-HPV, respectively. By cytology, dysplasia was not detected, but atypical squamous cells of undetermined significance (ASC-US) were diagnosed in two samples. No clinical change, suggestive of dysplasia/cancer, was detected.

**Conclusion:**

Our study was able to detect and characterize HPV infection by hybrid capture, which may represent a good tool for screening and follow-up of HPV in the studied population. The frequency and viral load of HPV were low. Neither clinical nor cytological changes suggestive of dysplasia/neoplasia were observed in oropharynx of HIV-positive patients.

## Introduction

HPV is a double-stranded DNA virus that is found in mucosa of the oral cavity, upper gastrointestinal tract and the anogenital tract (De Villiers et al., 2004). Nowadays, HPV infection is considered an important risk factor for oropharyngeal squamous cell carcinoma development (OSCC). Head and neck cancer is the sixth more prevalent worldwide and OSCC represents the most common malignancy in this region (Duvvuri & Myers, 2009). An epidemiological modelling study suggests that in 2020, HPV will cause more oropharyngeal than cervical cancers in the United States (Chaturvedi et al., 2011). There is also evidence showing that HPV-16 is the most prevalent type in HPV-related tumors and that the base of tongue and tonsils are usually the main affected areas (Gillisson et al., 2008).

The acquired immunodeficiency syndrome (AIDS) is a pandemic infection and represents one of the greatest public health concerns. The continuous improvement of antiretroviral therapy (ART) led to a better control and treatment of opportunistic infections with a decreased mortality rate and increased overall survival. Hence, a higher cancer incidence is expected in this population which requires the development of new prevention strategies (Bernstein et al., 2006). Viruses frequently associated with neoplasms in HIV patients include Kaposi’s sarcoma-associated herpesvirus (KSHV), Epstein-Barr virus (EBV) and HPV (Bernstein et al., 2006). Previous researches have investigated the association of HPV with head and neck cancer and also with dysplasia findings (Donà et al., 2014; D’Souza et al., 2007; Marks et al., 2014; Mehrotra, 2012; Migaldi et al., 2012; Mooij et al., 2014; Westra, 2014; Moyano et al., 2009). In spite of the clinical relevance of HPV infection in anogenital tract of HIV-positive patients, the relevance of oropharynx HPV infection in these patients is not clear. The aim of the present study was to detect HPV infection by different techniques (conventional vs liquid-based cytology, hybrid capture) and also clinical and cytological changes in oropharynx of HIV-positive patients.

## Methods

### Study design and eligibility

This observational study was conducted at University Hospital of Brasilia and approved by the Institutional Review Board (IRB from the University Hospital of Brasília—HUB/UnB/Approval number: 154.287) in accordance with the Declaration of Helsinki Principles. Written informed consent was obtained from all participants. In addition, the study was registered at the Brazilian Human Health Regulator for Research with the approval number CAAE 07802012.0.0000.5327 (Platform Brazil, aplicacao.saude.gov.br/plataformabrasil/login.jsf). Eligible criteria included (i) age ≥18 years; (ii) HIV infection diagnosis (positive serology confirmed by Western blot test).

### Demographic and clinical assessment

All patients agreed to participate using a semi-structured questionnaire. Data related to demographic characteristics (age and gender), sexual behavior (sexual practice/sexual orientation, condom use, oral intercourse, anal intercourse, number of sexual partners/year), lifestyle (current cigarette smoking or alcohol and other illicit drugs use e.g., cocaine, heroin, LSD), healthy status (oropharynx lesions, CD4+ cell counts, infection time, adherence to ART and previous sexually transmitted disease) were collected. In relation to sexually transmitted diseases (STDs), subjects were asked about genital and oral ulcerative or warts lesions which were suggestive of these diseases.

Subjects were initially submitted to a clinical oral examination to investigate the presence of pre-malignant lesions and cancer (i.e., leukoplakia, erythroplakia, ulcerous lesions). The samples were collected in triplicates from the oropharynx region (uvula, tonsils, soft palate and base of tongue). The collection technique involved back-and-forth movements in oropharyngeal mucosa. The smears were immediately fixed in 95% ethanol and stained by the Papanicolaou method. Samples were further assessed by conventional cytology, liquid-based cytology and hybrid capture assays.

### HPV genotyping

HPV genotype was assessed by hybrid capture (digene HC2 HPV DNA, QIAGEN, USA) able to identify 13 high-risk HPV subtypes (16, 18, 31, 33, 35, 39, 45, 51, 52, 56, 58, 59, 68) and 5 low-risk HPV types (6, 11, 42, 43, 44). The ratio relative light unit (RLU)/cutoff (CO)>1.0 was considered positive.

### Cytopathological evaluation

Conventional and base-liquid (ThinPrep PAP Test Kit; Cooper Surgical, Trumbull, CT, USA) cytology was used to assess the cytopathological changes. The cytological slides were examined by two trained and experienced pathologists without the knowledge of each other‘s diagnosis. In cases of non-concordance, the slides of reexamined to achieve a consensus.

The cytological findings were classified according to the current Bethesda system into the following categories: (i) negative, (ii) atypical squamous cells of undetermined significance (ASC-US), (iii) atypical glandular cells (iv) atypical squamous cells-cannot exclude high-grade intraepithelial lesion (ASC-H) (v) low-grade squamous intraepithelial lesion, (vi) high grade intraepithelial lesion and (vi) squamous cell carcinoma (Solomon, 2015). *Candida sp.* was diagnosed when pseudo-hyphae and/or small spores were present. Coccobacilli, characterized by small bacilli and cocci organisms, was considered as both isolated and microcolonies appearance. Finally, *Actinomyces sp* was diagnosed considering its filamentous morphological aspect.

### Statistical analysis

Statistical analysis was performed with Graphpad Prism 4 (GraphPad Software, San Diego, CA, USA) and SPSS1.3 software (Statsoft Inc, Tulsa, OK, USA). The contingence tables used *χ*2 and the Fisher test to analyze the different variables. Regarding missing data, this issue was addressed using completer’s only method. Statistical significance was assigned to *p* < 0.05.

## Results

### Demographical data and clinical findings

The demographic characteristics are listed in Table 1. Clinical oropharynx mucosa changes were present in 24% of the patients and they were mainly represented by edema (*n* = 6), hyperemia (*n* = 5), leucoplakias (*n* = 3) and tonsillar hyperplasia (*n* = 3).

**Table 1:** Clinical features of the HIV positive subjects.

Characteristics	No	(%)
**Gender**		
Men	68	68 (68/100)
Women	32	32 (32/100)
**Age (yrs old)**		
20–29	13	13 (13/100)
30–39	21	21 (21/100)
40–49	39	39 (39/100)
50–59	23	23 (23/100)
60–69	4	4 (4/100)
**Oropharynx lesion**		
Present	24	24 (24/100)
Absent	76	76 (76/100)
**CD4**^+^**lymphocyte count/mm**^**3**^		
<200	15	15.6 (15/96)
200–500	41	42.7 (41/96)
>500	40	41.7 (40/96)
**Duration of HIV infection (years)**		
≤1	15	15.2 (15/99)
2–5	21	21.2 (21/99)
6–10	25	25.3 (25/99)
>10	38	38.4 (38/99)
**Adherence to ART**		
Yes	88	88 (88/100)
No	12	12 (12/100)
**Tobaco smoking (current)**		
Yes	36	36 (36/100)
No	64	64 (64/100)
**Alcohol use (currente)**		
Yes	64	64 (64/100)
No	36	36 (36/100)
**Marijuana consumption**		
Yes	17	17 (17/100)
No	83	83 (83/100)
**Use of others illicit drugs**		
Yes	10	10 (10/100)
No	90	90 (90/100)
**Oral intercourse**		
Yes	64	65.3 (64/98)
No	34	34.7 (34/98)
**Anal intercourse**		
Yes	57	57.6 (57/99)
No	42	42.4 (42/99)
**Condom use**		
Yes	62	63.9 (62/97)
No	35	36.1 (35/97)
**Sexual orientation**		
Homosexual	40	40 (40/100)
Bisexual	40	40 (40/100)
Heterosexual	20	20 (20/100)
**Number of partners/yr**		
1	17	17.3 (17/99)
2–5	27	27.6 (27/99)
6–10	16	16.3 (16/99)
>10	38	38.8 (38/99)
**Previous history of STD**		
Yes	49	49 (49/100)
No	51	51 (51/100)
**Cytological features**		
Mild inflammatory	73	73 (73/100)
Moderate inflamatóry	18	18 (18/100)
Severe inflammatory	7	7 (7/100)
AS-CUS	2	2 (2/100)

### HPV infection

The amount of sample retrieved for the hybrid capture test was not enough to proceed with high and low risk HPV analysis in all sample size (*n* = 100). Therefore, high-risk HPV was investigated in 98 subjects and low-risk HPV in 48 subjects. HPV was detected in 16.3% (16/98) of the samples, with high and low risk HPV present in 8.2% (8/98) and 16.7% (8/48) samples, respectively. In samples with high and low-risk HPV the mean ± sd (maximum-minimum) of the ratio RLU/CO (viral load estimate) were respectively, 2.94 ± 2.58 (7.87–1.09) and 1.61 ± 0.65 (1.07–2.8). In two cases, high and low-risk HPV were simultaneously detected in the same sample.

**Table 2:** Variables and their association with HPV infection and profile (High and Low-risk).

	Human papillomavitus identification				High-risk HPV			Low-risk HPV		
	Non-infected (*n*%)	Infected (*n*%)	RC (IC 95%)	*P* value	*n* (%)	RC (IC 95%)	*P* value	*n* (%)	RC (IC95%)	*P* value
*Gender*										
*Men*	56 (82.4)	10 (14.7)	1.00	–	5 (7.3)	1.00	–	7 (10.3)	1.00	–
*Women*	28 (87.5)	4 (12.5)	0.80 (0.23–2.78)	0.965	3 (9.4)	1.26 (0.28–5.65)	1.000	1 (3.1)	0.33 (0.04–3.01)	0.561
*Age (yrs old)*										
20–29	10 (76.9)	3 (23.1)	1.00	–	2 (16.7)	1.00	–	2 (16.7)	1.00	–
30–39	18 (85.7)	3 (14.3)	0.55 (0.09–3.29)	0.849	2 (9.5)	0.58 (0.07–4.72)	1.000	1 (4.8)	0.28 (0.02–4.24)	0.778
40–49	33 (84.6)	6 (15.4)	0.61 (0.13–2.87)	0.832	3 (7.7)	0.46 (0.07–3.10)	0.786	4 (10.2)	0.40 (0.05–2.98)	0.732
50–59	20 (86.9)	2 (8.7)	0.33 (0.05–2.33)	0.520	1 (4.3)	0.26 (0.02–3.22)	0.630	1 (4.3)	0.29 (0.02–4.24)	0.778
60–69	3 (75.0)	0 (0.0)	–	0.918	0 (0.0)	–	1.000	0 (0.0)	–	1.000
*Oropharynnx lesion*										
Absent	66 (86.8)	9 (11.8)	1.00	–	4 (5.3)	1.00	–	5 (6.6)	1.00	–
Present	18 (75.0)	5 (20.8)	2.04 (0.61–6.84)	0.408	4 (16.7)	3.74 (0.85–16.34)	0.158	3 (12.5)	1.58 (0.32–7.76)	0.887
*TCD4+/mm*^3^										
<200	13 (86.7)	2 (13.3)	1.00	–	2 (13.3)	1.00	–	0 (0.0)	1.00	–
201–500	36 (87.8)	5 (12.2)	0.90 (0.16–5.24)	1.000	3 (7.3)	0.51 (0.07–3.42)	0.865	3 (7.3)	–	0.544
>500	31 (77.5)	7 (17.5)	1.47 (0.27–8.03)	0.969	3 (7.5)	0.56 (0.08–3.72)	0.929	5 (12.5)	–	0.220
*AVT*										
Não	8 (66.7)	4 (33.3)	1.00	–	3 (25.0)	1.00	–	2 (16.7)	1.00	–
Sim	76 (86.4)	10 (11.4)	0.26 (0.07–1.03)	0.116	5 (5.7)	0.18 (0.04–0.91)	0.087	6 (6.8)	0.64 (0.11–3.84)	1.000
*Tobacco smoking*										
Não	50 (78.1)	13 (20.3)	1.00	–	7 (10.9)	1.00	–	8 (12.5)	1.00	–
Sim	34 (94.4)	1 (2.7)	0.11 (0.014–0.91)	0.016	1 (2.7)	0.24 (0.03–1.99)	0.296	0 (0.0)	–	0.180
*Alcohol intake*										
Não	30 (83.3)	6 (16.7)	1.00	–	2 (5.6)	1.00	–	5 (13.9)	1.00	–
Sim	54 (84.4)	8 (12.5)	0.74 (0.24–2.34)	0.608	6 (9.4)	1.82 (0.35–9.54)	0.737	3 (4.7)	0.36 (0.07–1.73)	0.359
*Marijuana*										
Não	69 (83.1)	12 (14.4)	1.00	–	7 (8.4)	1.00	–	7 (8.4)	1.00	–
Sim	15 (88.2)	2 (11.8)	0.77 (0.15–3.79)	1.000	1 (5.9)	0.66 (0.08–5.75)	1.000	1 (5.9)	1.76 (0.16–19.48)	1.000
*Ilicit drugs*										
Não	74 (82.2)	14 (15.5)	1.00	–	8 (8.9)	1.00	–	8 (8.9)	1.00	–
Sim	10 (100.0)	0 (0.0)	–	0.376	0 (0.0)	–	0.700	0 (0.0)	–	1.000
*Oral intercourse*										
Não	29 (85.3)	4 (11.8)	1.00	–	2 (5.9)	1.00	–	2 (5.9)	1.00	–
Sim	53 (82.8)	10 (15.6)	1.37 (0.39–4.75)	0.849	6 (9.4)	1.63 (0.31–8.57)	0.846	6 (9.4)	2.71 (0.49–15.10)	0.435
*Anal intercourse*										
Não	36 (85.7)	5 (11.9)	1.00	–	2 (4.8)	1.00	–	3 (7.1)	1.00	–
Sim	47 (82.4)	9 (15.8)	1.38 (0.43–4.47)	0.592	6 (10.5)	2.34 (0.45–12.24)	0.510	5 (8.8)	1.67 (0.35–7.93)	0.796
*Sexual practice (men)*										
Homo	18 (85.7)	3 (14.3)	1.00	–	2 (9.5)	1.00	–	2 (9.5)	1.00	–
Heter	23 (85.2)	3 (11.1)	0.78 (0.14–4.35)	1.000	1 (3.7)	0.38 (0.03–4.50)	0.848	2 (7.4)	0.4 (0.04–3.52)	0.797
BI	15 (75.0)	4 (20.0)	1.60 (0.31–8.33)	0.884	2 (10.0)	1.12 (0.14–8.85)	1.000	3 (15.0)	1.28 (0.16–10.42)	1.000
*Sexual practice (women)*										
Homo	19 (100.0)	0 (0.0)	1.00	–	0 (0.0)	1.00	–	0 (0.0)	1.00	–
Heter	9 (69.2)	4 (30.8)	–	0.041	3 (23.1)	–	0.114	1 (7.7)	–	–
Bi	0 (0.0)	0 (0.0)	–	–	0 (0.0)	–	–	0 (0.0)	–	–
*Number of partners/yr*										
<1	23 (67.6)	10 (29.4)	1.00	–	7 (20.6)	1.00	–	5 (14.7)	1.00	–
1	11 (78.6)	3 (21.4)	0.63 (0.142.75)	0.791	1 (7.1)	0.29 (0.03–2.57)	0.454	2 (14.3)	1.60 (0.25–10.05)	1.000
>1	49 (96.1)	1 (1.9)	0.05 (0.01–0.39)	0.001	0 (0.0)	–	0.003	1 (2.0)	1.40 (0.13–15.26)	1.000
*Previous STDs*										
Não	43 (84.3)	8 (15.7)	1.00	–	5 (9.8)	1.00	–	5 (9.8)	1.00	–
Sim	41 (83.7)	6 (12.2)	0.79 (0.25–2.46)	0.680	3 (6.1)	0.63 (0.14–2.78)	0.804	3 (6.1)	0.90 (0.19–4.30)	1.000

Clinical data related to HPV-infected and non-infected individuals are listed in Table 2.

Oropharyngeal HPV infection was not found in homosexual women (0/19), but it was present in 30.8% (4/13) of heterosexual women (*p* = 0.041, Fisher test). The frequency of oropharyngeal HPV infection was 14.3% (3/21) in homosexual men, 11.1% (3/27) in heterosexual men and 20% (4/20) in bisexual men. These results did not show statistically significant differences. HPV infection was higher (*p* = 0.001) in individuals with more than 1 partner and significantly lower in smokers (*p* = 0.016). There was no association between oropharyngeal HPV infection and condom use, oral or anal intercourse, presence of oral lesions, CD4+ count cells, infection time and presence of DSTs.

### Cytopathological evaluation

**Figure 1:**
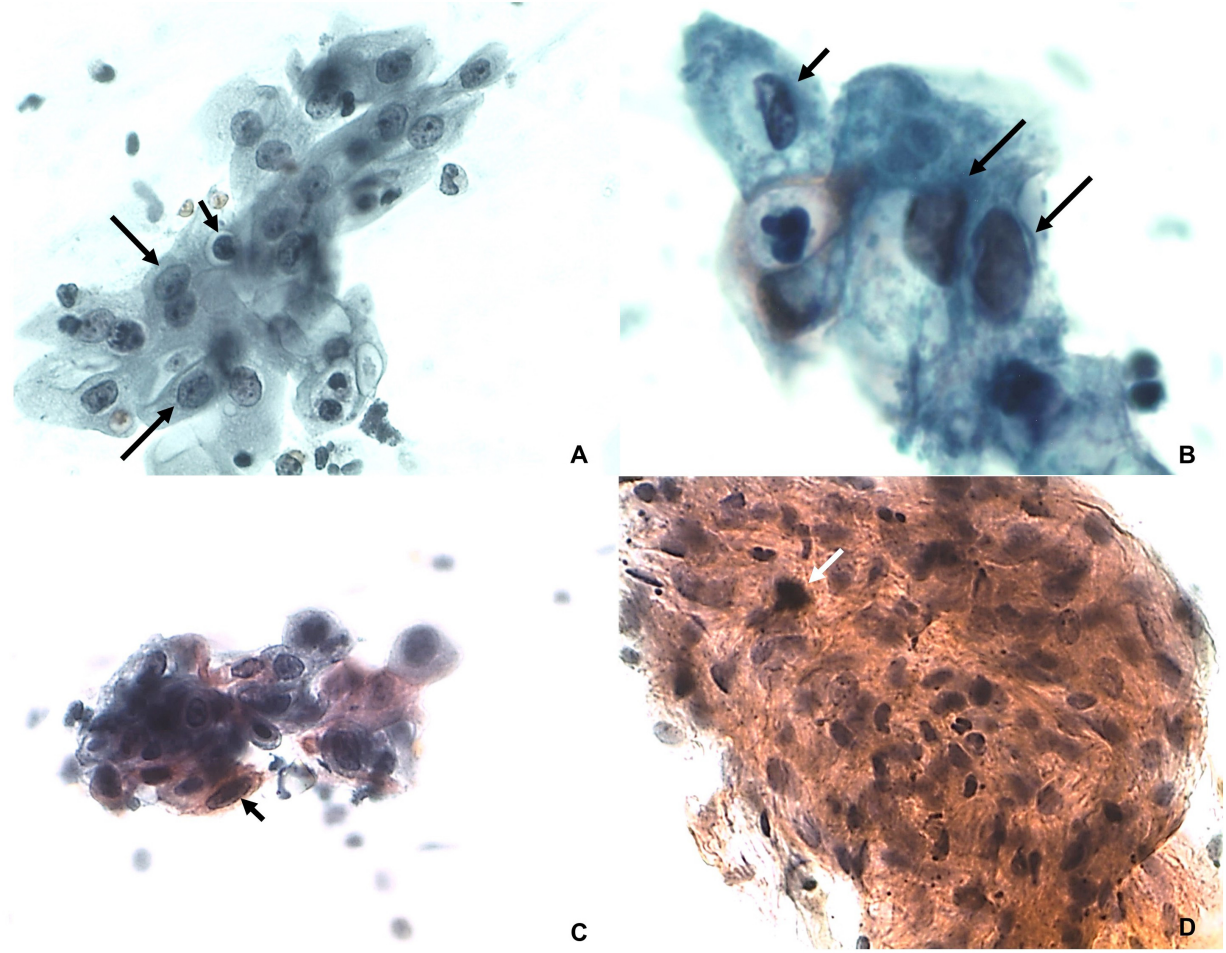
ASC-US. (A) Karyomegaly (arrows) and nuclear hyperchromatism (small arrow). Papanicolaou 100×. (B) Alteration of nucleus/ cytoplasm ratio (arrows) and nuclear hyperchromatism (small arrow). Papanicolaou 400×. (C) Nuclear pleomorphism with keratinized cells (arrow). Papanicolaou 100×. (D) Set of cells with intense parakeratinization and nuclear atypia (white arrow). Papanicolaou 100×.

**Figure 2:**
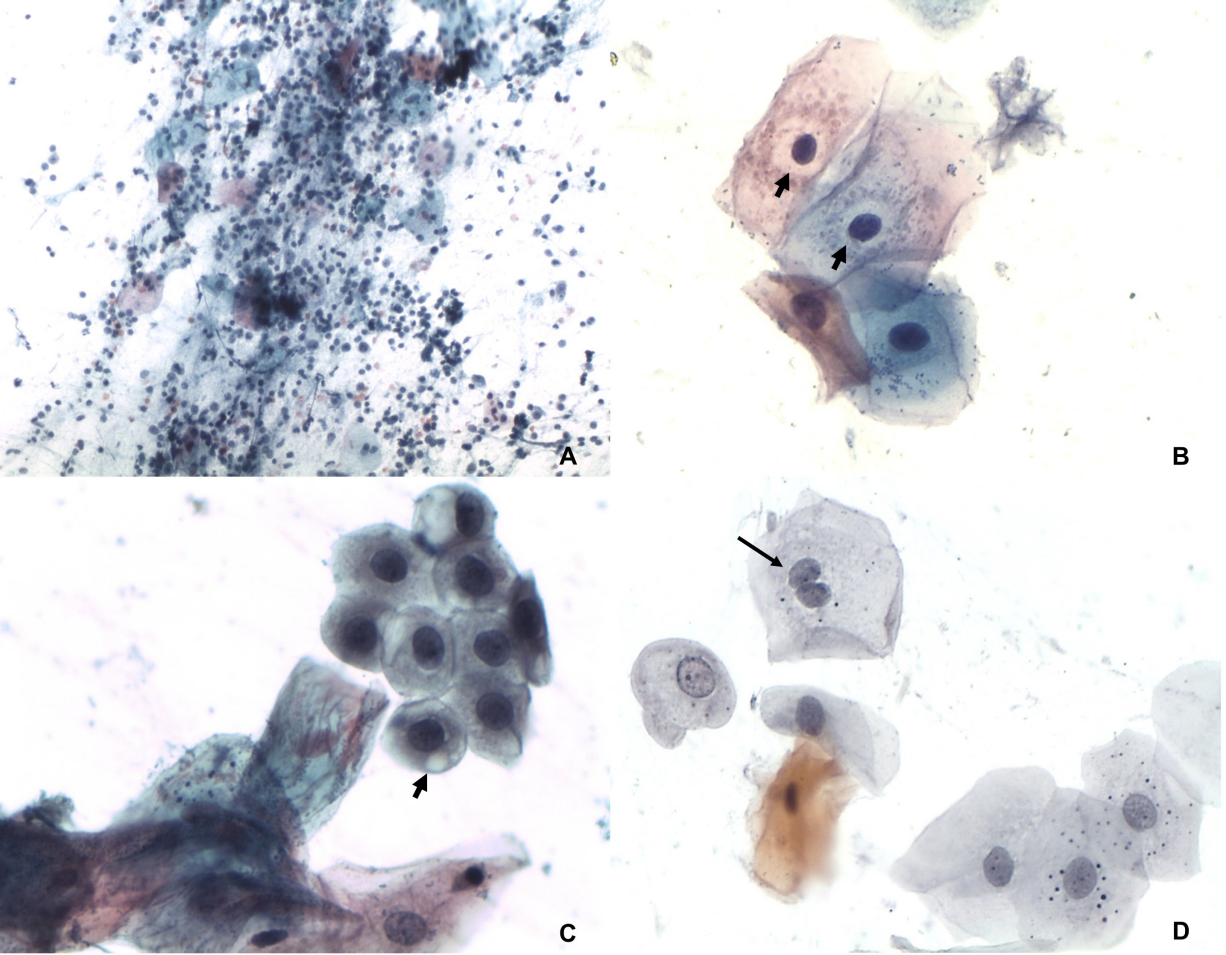
Inflammatory features. (A) Smear diffuse and intense inflammatory infiltrate composed by neutrophils and lymphocytes. Papanicolaou 100×. (B) Perinuclear transparent zone (arrows). Papanicolaou 400×. (C) Cytoplasmic vacuolation (arrow). Papanicolaou 400×. (D) Binucleation (arrows) Papanicolaou 400×.

The cytopathological evaluation revealed 98 negative and two ASC-US samples (Fig. 1). The inflammatory features were characterized by presence of leukocytes, perinuclear transparent zone, cytoplasmic vacuolation and binucleation (Fig. 2). The microflora was represented by *Candida sp. Actinomyces* sp, cocci and bacilli. The analysis performed on liquid-based cytology showed only inflammatory changes in all samples.

## Discussion

The present study analyzed the possible association between demographic characteristics, clinical assessment and cytopathological features with HPV infection in oropharyngeal samples of HIV-positive subjects. To our knowledge, the current work represents the first study in which oropharyngeal mucosa of HIV-positive individuals was simultaneously analyzed by conventional, liquid-based cytology and hybrid capture for HPV and pre-malignant and malignant detection.

It is important to mention that oral and genital HPV is more prevalent in HIV-positive individuals compared to HIV-negative (Robbins et al., 2015; Videla et al., 2013). In a prospective cohort of HIV-positive men, Bernstein et al. (2006) showed that HPV infection is less prevalent in oral region than anal and penile sites.

Regarding to the methods of HPV detection, most of studies used the polymerase chain reaction (PCR) to analyze HPV infection in oropharynx (Chaturvedi et al., 2011; Migaldi et al., 2012; Mooij et al., 2014; Westra, 2014; Moyano et al., 2009; Robbins et al., 2015). There is a great variability of HPV infection rate in oropharyngeal mucosa of HIV subjects, ranging from 4.7% to 61.3% (Donà et al., 2014; Robbins et al., 2015; Beachler et al., 2013; Bernstein et al., 2006; Gaester et al., 2014; Parisi et al., 2011; Read et al., 2012; Fakhry et al., 2011). In the current study, HPV’s DNA was found in 16.3% (16/98) of oropharyngeal mucosa samples. Such variability can be attributed to differences in sample collection, number of HPV types tested, participants’ characteristics and DNA detection methods. The use of Hybrid Capture may be a cause of variability in present study. Hybrid Capture is routinely used to detect HPV in the cervix. In contrast, the examination of oropharynx mucosa by this test is not routinely used. Hybrid Capture can be defined as a sensitive test for HPV detection. It is able to recognize all thirteen HPV high-risk genotypes classified as class I carcinogenic with respect to cervical cancer by the WHO (Cogliano et al., 2005). Although quantitative PCR is the standard measurement to detect the viral load, the HC test has also been approved by the US Food and Drug Administration (FDA) for cervical samples. It should be noted, however, that it has not been validated as a quantitative test, in spite of the fact that RLU/CO values have been considered as a good estimation of the HPV load (Terry et al., 2001; Meijer et al., 2009).

Regarding to methods of sample collection, many procedures are reported for cell collection such as mouth rinsing, saliva collection, scrape and cytobrush (Donà et al., 2014; Marks et al., 2014; Beachler et al., 2013; Fakhry et al., 2011; Fuller et al., 2015; Marques et al., 2013). In our view, scraping with flexible brush allows better sample collection from a specific anatomic location (oropharyngeal mucosa-posterior and anterior pharyngeal walls, tonsils, soft palate, and tongue base). In addition, the material collected by scrape has a higher number of cells and is more representative (Donà et al., 2014).

Oral sex and open mouthed-kissing have been associated with oral HPV infection in normal and HIV positive individuals and a protected sexual intercourse is the most important factor to avoid HPV oral infection (Beachler et al., 2013; Gaester et al., 2014). Nonetheless, no association was found between condom use and oral sex with oropharyngeal HPV infection in the current study. Regarding sexual orientation, some studies reported a higher frequency of HPV infection in heterosexual individuals and bisexual women (Videla et al., 2013; Ragin et al., 2011). The findings of the present study also detected a higher HPV prevalence in heterosexual women.

We detected a negative association between oropharyngeal HPV infection and cigarette smoking. It is known that tobacco consumption increases epithelial thickness (Villar & Lima, 2003; Kumar & Faizuddin, 2011). Therefore, it can be hypothesized that a thicker oral epithelium may lead to a protective effect against HPV infection. Moreover, no significant association with previous history of recurrent alcohol and illicit drug use was found in present study.

Clinical mucosa lesions were detected in 24% of patients. Oropharyngeal HPV infection was present in five patients with lesions, but no association between presence of oropharynx lesions and oropharynx HPV infection was detected. This can be justified because most of these lesions were represented by hyperemia and not by leuko or erytroplakia.

Bethesda System is used for classification of cervical and anal malignant and pre-malignant lesions in cytology. Considering that there is a lack of a classification system for HPV related- and malignant and pre-malignant lesions in oral cytology, Bethesda classification was chosen in the current study to proceed the cytological analysis. Such classification has already been used by some studies to classify oral cytological findings (Solomon, 2015; Fakhry et al., 2011; Dalstein et al., 2003; Navone, 2009; Maia et al., 2016). By conventional cytology, the cytological smears analyzed showed good cellularity and the majority were negative (98%); ASC-US was diagnosed two cases. Koilocitosis, a marker of nuclear changes consistent with HPV infection, was not detected in any sample.

At to date, there are no studies investigating the association between intraepithelial lesion/dysplasia and HPV viral load in oropharynx. On cervical and anal samples, high viral loads are associated with a greater chance of malignant progression than low viral loads (Dalstein et al., 2003). Among the samples classified as ASC-US in the present study, HPV was detected in just one with viral load estimate of 2.68 and 1.30 for high and low risk, respectively. In the other ASC-US sample it is possible that atypical cells were present as a consequence of *Candida sp* infection.

The present study should be considered as a preliminary study and some limitations must be mentioned. First, the number of samples (*n* = 100) is relatively small. The samples were collected in triplicates from the oropharynx region to be assessed by conventional cytology, base-liquid cytology and hybrid capture assays. However, the volume of the samples provided for the hybrid capture test were not sufficient for the application of the high- and low-risk HPV kits in all 100 patients. High-risk HPV was investigated in 98 samples and low-risk HPV in 48 samples. It was not possible to compare the results of HPV status in oropharynx between healthy and HIV-positive individuals. A control group age and gender-matched would be feasible. However, several important variables would be very difficult to control, such as number of sexual partners, use of illicit drugs, alcohol intake and etc. Besides, the clinical course of HIV and HPV would certainly have interactions that would not be present in healthy subjects. Interestingly, some of such data in the healthy population have already been described previously (Beachler et al., 2013). In addition, further studies with a larger population group using a more thorough and in-depth questionnaire, larger genotyping detection and long follow-up should be undertaken to better evaluate the development and progression of oral HPV lesions in this population. Finally, a possible correlation between HPV load and progression to dysplasia in oropharynx seems interesting and should be further investigated. It should be considered as a preliminary study and further work could be considered.

## Conclusions

The use of hybrid capture was able to detect and characterize HPV infection; hence, this method may represent a good tool for screening and follow-up of this population. The HPV frequency and load viral were low, and neither clinical nor cytological changes suggestive of dysplasia/neoplasia were observed in oropharynx of HIV-positive subjects. In our study, the absence of lesions could be attributed to the low HPV viral load. Therefore, a careful follow-up of patients with ASC-US and HPV infection is recommended to evaluate possible evolution to dysplasia and/or development of others oral lesions.

##  Supplemental Information

10.7717/peerj.4407/supp-1 Supplemental Information 1 Raw data HPV oropharynx

10.7717/peerj.4407/supp-2 Supplemental Information 2 Questionnaire

## Additional Information and Declarations

### Data Availability

The following information was supplied regarding data availability: The raw data has been provided as a Supplemental File.
