# Supplementary material for: Oropharynx HPV status and its relation to HIV infection
Source: PeerJ. 2018 Mar 22;6:e4407. doi: 10.7717/peerj.4407 (PMC5866915; doi:10.7717/peerj.4407)
Supplement: Supplemental Information 2 [file peerj-06-4407-s002.doc]

**QUESTIONÁRIO**

**NOME:**

1- Sexo: ( ) feminino ( ) masculino

2- Idade: ____anos

3- Lesão oral externa ( ) sim ( ) não

4- Estado imunitário (dosagem de CD4): ______/mm³

(dosagem CD8):________/mm^3^

CD4/CD8_____________

Carga viral cópias:___________

Log:_____________

5- Tempo de infecção pelo HIV: _________anos

6- Uso de terapia ARV: ( ) sim ( ) não

7- Prática coito anal: ( ) sim ( ) não

8- Prática coito oral:( ) sim ( ) nao

9- Quando iniciou o coito anal: ________ anos

10-Quando iniciou o coito oral:_______ anos

11- Número de parceiros:_____________

12-Tabagismo: ( ) sim ( ) não Qtos cigarros/dia?:

13- Etilismo: ( ) sim ( ) não Bebe qtos dias na semana?:

14- Usou maconha: ( ) sim ( )não

15- Uso de drogas: ( ) sim ( ) não

16- Parceiro(a) atual com lesão anogenital: ( )sim ( )não

17- Parceiro(a) atual com lesão anogenital oral: ( )sim ( )não

18- Parceiro(s) anterior(es) com lesão anogenital ou oral por HPV: ( ) sim ( ) não

19-Frequência das atividades sexuais

20-Usa preservativo: ( ) sim ( ) não

21- História de doença sexualmente transissível

22- Orientação sexual:

- - Heterosexual ( )
  - Homossexual ( )
  - Bissexual ( )

23 – DATA:
